# Global gene expression profiling identifies new therapeutic targets in acute Kawasaki disease

**DOI:** 10.1186/s13073-014-0102-6

**Published:** 2014-11-20

**Authors:** Long Truong Hoang, Chisato Shimizu, Ling Ling, Ahmad Nazri Mohamed Naim, Chiea Chuen Khor, Adriana H Tremoulet, Victoria Wright, Michael Levin, Martin L Hibberd, Jane C Burns

**Affiliations:** Genome Institute of Singapore, Singapore City, Singapore; Department of Pediatrics, University of California San Diego and Rady Children’s Hospital, La Jolla, CA 92093 USA; Section for Pediatrics, Division of Medicine, Imperial College, London, UK

## Abstract

**Background:**

Global gene expression profiling can provide insight into the underlying pathophysiology of disease processes. Kawasaki disease (KD) is an acute, self-limited vasculitis whose etiology remains unknown. Although the clinical illness shares certain features with other pediatric infectious diseases, the occurrence of coronary artery aneurysms in 25% of untreated patients is unique to KD.

**Methods:**

To gain further insight into the molecular mechanisms underlying KD, we investigated the acute and convalescent whole blood transcriptional profiles of 146 KD subjects and compared them with the transcriptional profiles of pediatric patients with confirmed bacterial or viral infection, and with healthy control children. We also investigated the transcript abundance in patients with different intravenous immunoglobulin treatment responses and different coronary artery outcomes.

**Results:**

The overwhelming signature for acute KD involved signaling pathways of the innate immune system. Comparison with other acute pediatric infections highlighted the importance of pathways involved in cell motility including paxillin, relaxin, actin, integrins, and matrix metalloproteinases. Most importantly, the IL1β pathway was identified as a potential therapeutic target.

**Conclusion:**

Our study revealed the importance of the IL-1 signaling pathway and a prominent signature of innate immunity and cell migration in the acute phase of the illness.

**Electronic supplementary material:**

The online version of this article (doi:10.1186/s13073-014-0102-6) contains supplementary material, which is available to authorized users.

## Introduction

Kawasaki disease (KD) is a self-limited vasculitis of unknown etiology that predominantly affects children aged younger than 5 years [1]. The incidence of the disease varies widely among different populations from a high of 240 in Japan to five in Norway per 100,000 children aged under 5 years [2,3]. According to the current paradigm, KD is an inflammatory process triggered in genetically susceptible children following exposure to a stimulus that may be a common antigen or infectious agent. The inflammation associated with KD affects the arterial wall and leads to coronary artery aneurysms (CAA) in 25% of untreated KD children [4], making KD the most common cause of acquired heart disease in children in developed countries [5]. Timely diagnosis is critical for treatment with intravenous immunoglobulin (IVIG) to be effective in reducing aneurysm rates to approximately 5% [6]. However, IVIG resistance, defined as the persistence or recrudescence of fever, has been widely reported with rates varying from 10% to 30%, and these patients are at higher risk of CAA formation [6–8]. Previous studies have examined gene expression profiles and described KD-specific signatures, but these studies have had limited power due to small sample size [9,10]. The present study of a large KD cohort defines the global gene expression signatures of acute KD, aneurysm formation, and resistance to therapy with the identification of potential new therapeutic targets.

## Methods

### Subjects

Kawasaki disease: patients diagnosed with KD had fever for at least 3 days but not more than 10 days, and met at least four of five clinical criteria for KD (rash, conjunctival injection, cervical lymphadenopathy, oral mucosal changes, and changes in the extremities) or three of five criteria and coronary artery abnormalities documented by echocardiogram [6]. Whole blood RNA was collected in PAXgene tubes during the acute phase, prior to IVIG administration, from 146 KD subjects, and after the resolution of the acute illness and after the erythrocyte sedimentation rate (ESR) decreased to <40 mm/h and the C-reactive protein (CRP) level decreased to <1.0 mg/dl (convalescent phase, illness day 19 to 2,230) in 131 subjects. (Additional file 1: Figure S1A) Complete blood counts and other clinical laboratory testing were performed on the same blood sample used for transcript analysis. Coronary artery dimensions were described by the variable Z_max_, which was defined as the maximal Z score (standard deviation units from the mean) of the internal diameter of the left anterior descending and right coronary arteries normalized for body surface area during the first 6 weeks after illness onset. IVIG treatment resistance was defined as persistent or recrudescent fever at least 36 h after the end of their IVIG infusion. All patients were enrolled at Rady Children’s Hospital San Diego after obtaining written parental informed consent and patient assent as appropriate. The study protocol was conducted in accordance with the declaration of Helsinki and reviewed and approved by the University of California - San Diego Institutional Review Board.

### Gene expression microarray

RNA expression was analyzed according to the detailed protocol as previously published [11]. In brief, whole blood (2.5 mL) was collected directly into PAXgene RNA tubes (Qiagen, Sussex, UK). RNA extraction was performed using Paxgene RNA kits (Qiagen). Biotinylated amplified cRNA was generated by *in vitro* transcription (IVT) technology using Illumina TotalPrep RNA Amplification Kit (Ambion, Inc., Austin, TX, USA) according to the manufacturer’s instructions. After purification, 2 μg of cRNA was hybridized to an Illumina HumanRef-12 V4 BeadChip (containing probes for more than 47,000 gene transcripts) at 55°C for 18 h following the manufacturer’s instructions (Illumina, Inc., San Diego, CA, USA). This was followed by washing, blocking, and streptavidin-Cy3 staining steps. Finally, the chip was scanned with an Illumina Bead Array Reader confocal scanner and checked using Illumina QC analysis. Background subtracted raw gene expression intensity data were exported from Genome studio and used for further analysis. All the raw and normalized gene expression data are available at the GEO public database. The accession number is GSE63881.

### Validation by reverse transcriptase-polymerase chain reaction

To validate the microarray results, transcript abundance levels were measured by reverse transcriptase-polymerase chain reaction (RTPCR) for *IL1B* (ABI, Hs01555410_m1), *IL1R1* (ABI, Hs00991002_m1), *IL1R2* (ABI, Hs01030384_m1), *IL1RAP* (ABI, Hs00895050_m1), and *IL1RN* (ABI, Hs00893626_m1), for a new patient cohort of 20 KD subjects using acute and convalescent paired whole blood RNA samples (PAXgene tubes). Controls (n = 10) were age-similar healthy children undergoing minor elective surgery (Additional file 2: Table S5). Relative abundance of the target transcripts was normalized to the expression level of the housekeeping gene, TATA box-binding protein-associated factor, RNA polymerase I, B (*TAF1B*)*,* as previously described [10].

### Reference datasets

Gene expression data (raw data) from whole blood from children with confirmed bacterial infections, viral infections, and healthy controls were downloaded from published papers. From GSE40396 study [12], we retrieved gene expression data for 22 healthy children who were afebrile and tested negative for viral infections, and from eight, 11, six, 10, and eight children infected with bacteria, adenovirus, enterovirus, human herpes virus (HHV)-6 virus, and rhinovirus, respectively. Expression data from children with adenovirus, enterovirus, and HHV-6 were combined to form a pan-virus group of 27 patients. Expression profiles from rhinovirus-infected patients were not included in the analysis because these patients were afebrile when the samples were collected and rhinovirus is not generally associated with systemic signs of inflammation including fever. (Additional file 1: Figure S1B). From dataset GSE42026 [13], we retrieved expression data for 33 healthy pediatric control subjects, 18 subjects with Gram-positive bacterial infection (of whom five were co-infected with viruses), 19 subjects with influenza 09/H1N1 infection only, and 22 subjects infected with respiratory syncytial virus (RSV) only (Additional file 1: Figure S1C).

### Data normalization

All datasets were normalized by using R [14]. First, the raw data were log10 transformed before Z score transformation was performed [15]. Z score was calculated within each sample by subtracting the overall mean gene intensity from the raw intensity signal for each gene. After that, these data were divided for the standard deviation of all of the measured intensities using the following formula:

$$ \mathrm{Z}\;\mathrm{score}=\frac{\mathrm{intensity}\;\mathrm{G}\kern0.24em \hbox{-} \kern0.24em \mathrm{mean}\;\mathrm{intensity}\;\mathrm{G}1\dots \mathrm{G}\mathrm{n}\;}{\mathrm{SD}\;\left(\mathrm{G}1\dots \mathrm{G}\mathrm{n}\right)} $$

Where G is any gene on the microarray and G1…Gn represents the aggregate measure of all of the genes.

### Statistical analysis

We used Z score (standard deviation units from the mean) as the base value to identify differentially abundant transcripts (DATs) in comparisons between any two groups of samples. Transcripts with high Z scores were those that were more abundant while those with low Z scores were the less abundant [15]. Conventional fold-change calculations were not used because at low intensities, when data are much more variable, the false discovery rate increases. To identify DATs in each group of patients, a Z-score ratio for each gene was calculated [16]. The Z-score ratio was calculated by dividing the mean difference in Z score between the groups by the standard deviation of the Z score difference across all the genes.

$$ \mathrm{Z}\;\mathrm{ratio}=\frac{\mathrm{Mean}\;\mathrm{Z}\;\mathrm{score}\;\left(\mathrm{group}\;1\right)-\mathrm{mean}\;\mathrm{Z}\;\mathrm{score}\;\left(\mathrm{group}\;2\right)}{\mathrm{SD}\;\mathrm{of}\;\mathrm{Z}\;\mathrm{score}\;\mathrm{differences}\;\mathrm{G}1\dots \mathrm{G}\mathrm{n}} $$

Where G1…Gn represents the aggregate measure of all the genes. A Z ratio of ±1.96 is equivalent to the significance level of *P* <0.05 [15].

The Z test was used as an additional method for identification of DATs where δ^2^ is the standard deviation of gene i in group1 or group 2, n is the sample size in each group.

$$ \mathrm{Z}\;\mathrm{test}=\frac{\mathrm{Mean}\;\mathrm{Z}\;\mathrm{score}\;\left(\mathrm{group}\;1\right)-\mathrm{mean}\;\mathrm{Z}\;\mathrm{score}\;\left(\mathrm{group}\;2\right)}{\sqrt{\frac{\updelta^2}{{\mathrm{n}}_1}+\frac{\updelta^2}{{\mathrm{n}}_2}}} $$

DATs were defined as follows: (1) a Z ratio of ±1.96; and (2) an adjusted Z test *P* value of ≤0.05 and expressed in at least one sample.

### Generalized linear model (GLM)

Because the mean yield of total RNA in acute samples were significantly higher than in the convalescent samples, probably due to the differences in the total number of white blood cell counts in the blood samples, we decided to use absolute cell number in acute and convalescent KD subjects as a covariant in a generalized linear model (GLM) when comparing their transcriptome profiles.

$$ \mathrm{G}\mathrm{i}\mathrm{j}=\upbeta 0+\upbeta 1\mathrm{Covariates}+\upbeta 2\mathrm{Group} $$

Where Gij denotes transformed-Z score normalized expression value for individual i at day j; Covariates were the absolute number of lymphocytes, neutrophils and monocytes and Group was either 1 for acute or 0 for convalescent. Benjamini-Hochberg multiple testing correction was applied. DAT were defined to have corrected *P* value <0.05 and fold-difference >1.5.

### Ingenuity pathway analysis

DATs were analyzed by ingenuity pathway analysis (IPA) [17]. The IPA database contains canonical pathways and functional gene relationships expertly curated from the literature which helps in understanding disease processes by identifying key biological functions and novel molecular networks. DAT lists were cross-referenced against this database to identify enriched pathways associated with the clinical conditions. Significant canonical pathways were defined as having a Fisher’s exact test *P* value ≤0.05 (B-H correction).

### Summary of samples and datasets for analysis

We successfully analyzed 146 samples at the acute phase and 131 samples at the convalescent phase from the KD subjects. Of these 146 patients, 16 (11%) had coronary artery aneurysms (CAA), 30 (20.5%) had transiently dilated CA, and 100 (68.5%) had normal CA (Zmax <2.5). For the analysis of treatment response, the six patients treated with IVIG plus infliximab for cardiac indications were excluded. Of the remaining 140 patients, 110 (79%) were responsive to IVIG treatment and 30 (21%) were resistant. Of these 30 resistant subjects, 11 (30.5%) developed CAA (Additional file 1: Figure S1A).

#### Identification of differentially abundant transcripts

There were 39,390 probes that were common in all datasets and they were used for identification of DATs in each subject group.

#### Acute vs. convalescent KD subjects

The baseline characteristics of these patients are summarized in Additional file 2: Table S1. Gene expression profiles from 146 acute KD patients were contrasted with those from 131 convalescent patients without taking into account the difference in the cell numbers between them. From the total of 39,390 probes, 2,414 were differentially expressed (adjusted *P* value ≤0.05, 1.96 < Z ratio < -1.96) between the acute and convalescent KD subjects. Of the 2,414 probes, 1,541 probes were more abundant 873 probes were less abundant in acute samples. Using the GLM model to take into account differences in peripheral blood cell numbers, we identified 1,083 DATs between acute and convalescent subjects. Among these 1,083 DATs, 264 were less abundant and 819 transcripts were more abundant in acute samples.

#### Infectious disease control subjects vs. healthy children

From the GSE40396 dataset, we identified 2,395 transcripts (adjusted *P* value ≤0.05, 1.96 < Z ratio < -1.96) that were more (1,053) or less (1,342) abundant in children infected with adenovirus, enterovirus, or HHV-6 in comparison with the healthy controls. Similarly, we identified 2,233 DATs (933 more and 1,300 less abundant) in bacteria-infected patients compared to healthy controls. In comparison with the healthy controls in GSE42026 dataset, we identified 2,175 (adjusted *P* value ≤0.05, 1.96 < Z ratio < -1.96) (1,064 more and 1,111 less abundant) DATs in influenza 09H1N1-infected patients, 1,823 (826 more, 987 less abundant) DATs in RSV-infected patients (adjusted *P* value ≤0.05, 1.96 < Z ratio < -1.96), and 2,327 DATs (1,007 more and 1,320 less abundant) in children infected with a bacterial pathogen (adjusted *P* value ≤0.05, 1.96 < Z ratio < -1.96).

## Results

### Gene ontology analysis

#### Acute vs. convalescent KD

**Figure 1:**
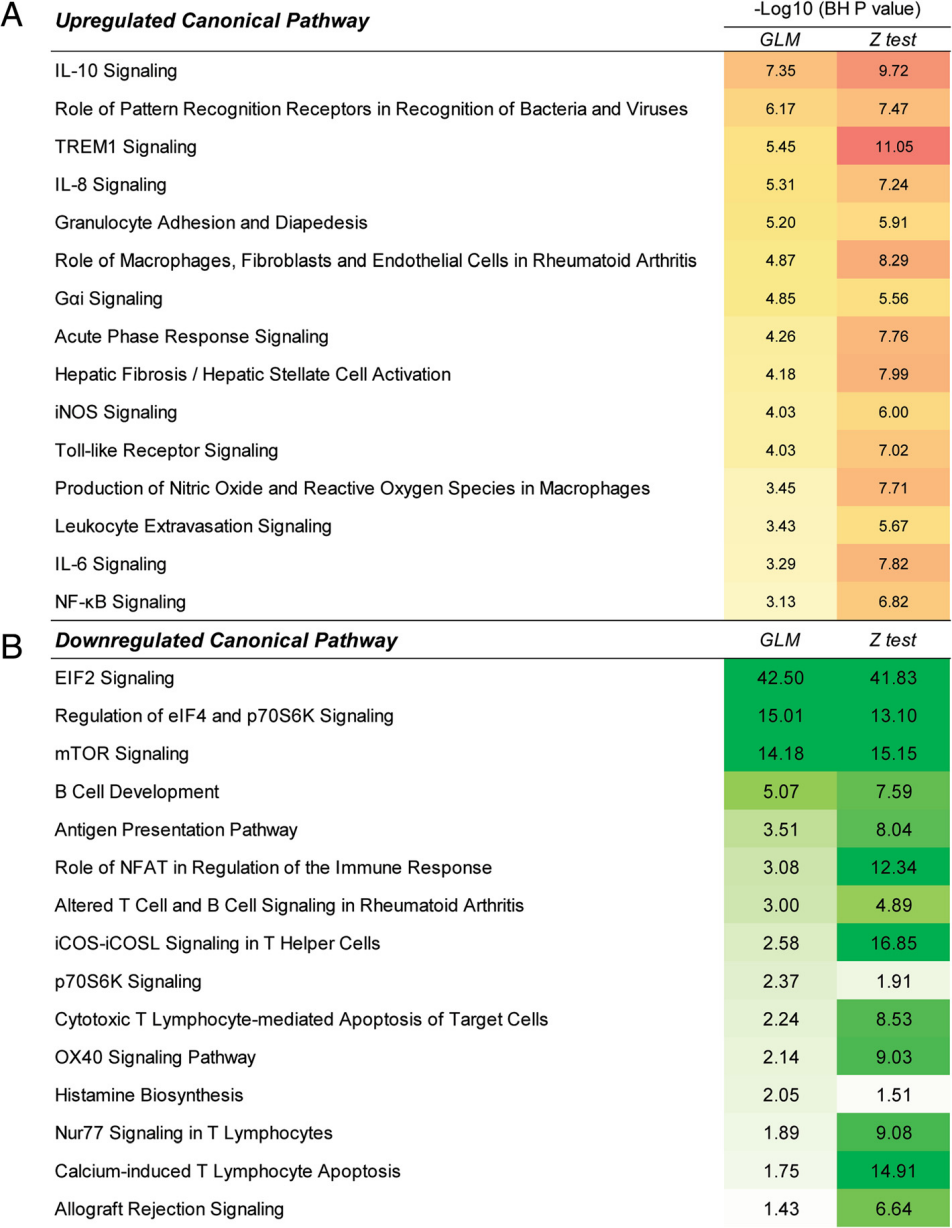
**Overlapping differentially expressed pathways in acute vs. convalescent KD determined by both GLM and by Z test. (A)** Top 15 upregulated pathways in acute KD were highly involved in innate responses; **(B)** 15 downregulated pathways in acute KD were highly involved in host protein synthesis and T cell responses. The *P* values were determined using Fisher’s Exact Test with Benjamini-Hochberg multiple testing corrections. Although there was a large difference in the number of DATs, the most significant pathways were highly similar between the two methods.

Because we had detailed clinical laboratory data from our KD subjects concurrent with the whole blood RNA samples, we assessed whether cell subtype numbers affected gene ontology. The difference in lymphocyte, neutrophil, and monocyte numbers between acute and convalescent samples were taken into account using the GLM model. Fewer DATs (1,083 vs. 2,414) were identified when cell numbers were taken into account and gene ontology analysis for the more abundant transcripts at acute stage (819 and 1,541) identified 110 and 183 significant pathways in the GLM and Z test, respectively. Among these pathways, 95 were common to both analyses. Gene ontology analysis for the less abundant transcripts at the acute stage (264 and 873) identified 17 and 47 significant pathways in the GLM and Z test, respectively, of which 15 pathways were common. Although there were significant differences in the numbers of DATs between the GLM and Z test methods, there was significant overlap between the key canonical pathways identified by either method. While the cell count numbers are likely to be proportional to any specific immune cell type, we cannot rule out the possibility that small specific subsets of immune cells may have a large effect on these results. However, the large overlap suggests that these effects are likely to be small. Prominent among the genes driving the common upregulated pathways (Figure 1A) were genes involved in IL-1 signaling and the innate immune response. Prominent among the common downregulated pathways (Figure 1B) were genes in the family encoding for ribosomal proteins and T-cell related genes including CD3, LCK, and HLA Class II antigens.

#### IVIG treatment response

**Figure 2:**
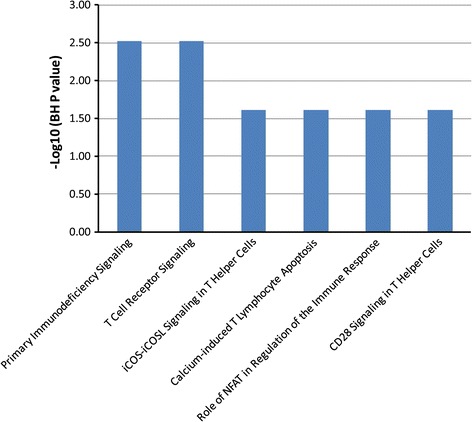
**Gene pathways that were up-regulated in patients who were responsive to IVIG treatment in comparison with IVIG-resistant patients.** DATs were derived from Z test. The Y axis depicts the -log10 of BH corrected *P* value which identified by Fisher’s exact test. The majority of the pathways were T cell, NK cell-related response.

The baseline characteristics of IVIG-responsive and resistant subjects are summarized in Additional file 2: Table S2. To identify transcripts that distinguish IVIG-responsive and IVIG-resistant subjects, we used the Z test to compare the acute expression profiles of subjects who were responsive (n = 110) and resistant to IVIG treatment (n = 30). We identified 137 transcripts that were differentially abundant between the IVIG-responsive and IVIG-resistant subjects. Pathway analysis for these 137 transcripts showed that most of the pathways were involved in T cell-related responses (Figure 2). Transcripts that were involved in these pathways included CD3E, CD4, ITGA5, ZAP70, NFkB2, LCK, PAFAH1B3, and TNFSF13B. When stratifying genes by the magnitude of the fold-difference, the top DATs were matrix metalloproteinase-8 (MMP-8), ankyrinD22, carcinoembryonic antigen cell adhesion molecule 1 (CEACAM1), Fructose-2,6 biphosphatase 2 (PFKB2), and haptoglobin (HP) with higher fold-difference in transcript abundance in subjects with resistance to IVIG (Additional file 2: Table S3).

#### Coronary artery outcomes

**Figure 3:**
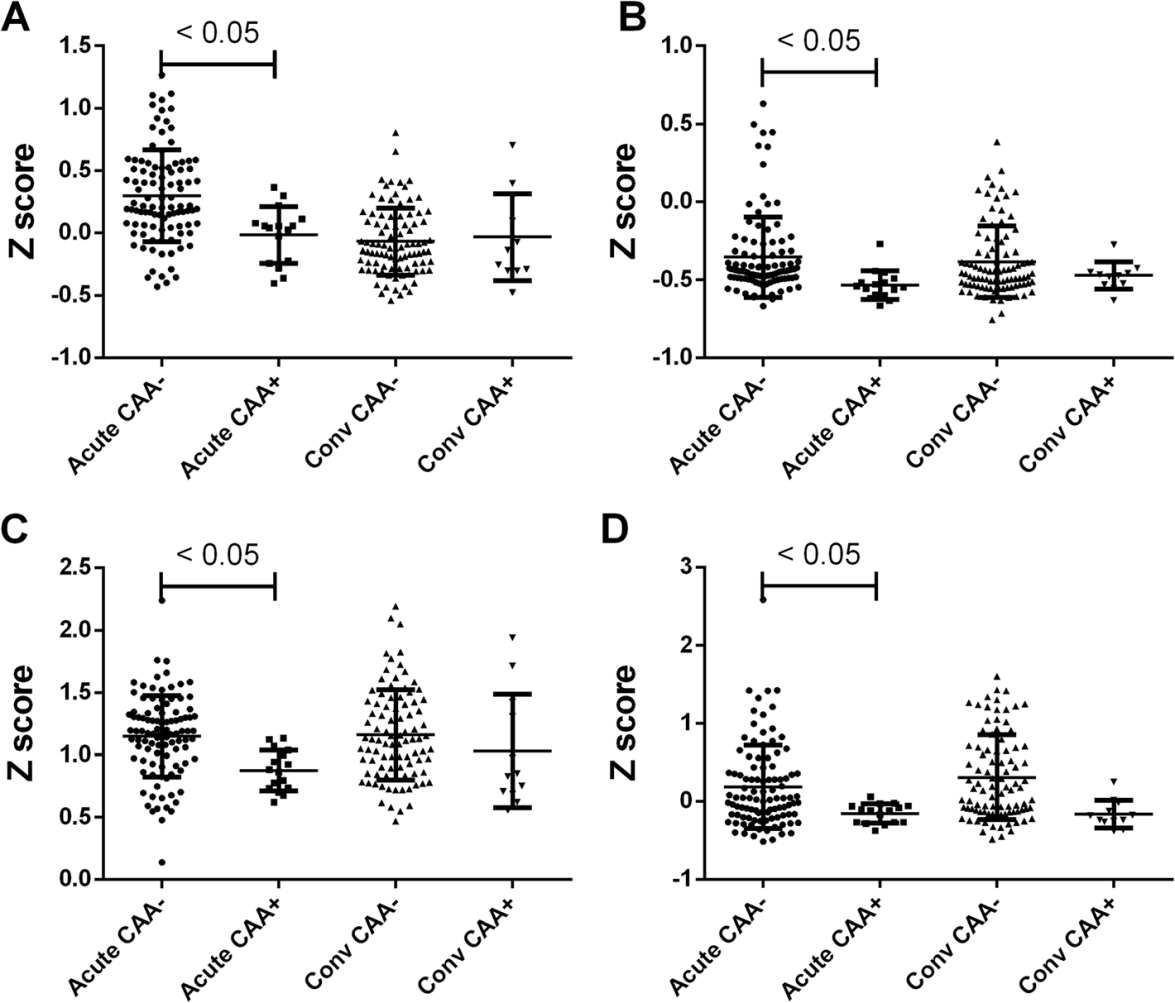
Comparison of transcripts from KD subjects with normal coronary arteries and those with coronary artery aneurysms by Z test revealed only four DATs: (A) ASPRV1, (B) CYP26B1, (C) TRANK1, and (D) NKX3-1 * ***P*** **value <0.05.**

The baseline characteristics of subjects with normal CA and with CAA are summarized in Additional file 2: Table S4. We chose to analyze only the extreme phenotypes of normal and CAA + without including the transiently dilated subjects. The Z test comparison between subjects with normal CA (n = 100) and those who developed CAA (n = 16) found only four DATs (ASPRV1, CYP26B1, TRANK1, and NKX3-1) which were all downregulated in CAA + compared to normal CA. The cytochrome P450 oxidase, CYP26B1, had the greatest fold-difference and was suppressed in subjects who developed coronary artery abnormalities compared to subjects with normal arteries (*P* = 0.003) (Figure 3).

#### Comparison of KD with pediatric infectious diseases

**Figure 4:**
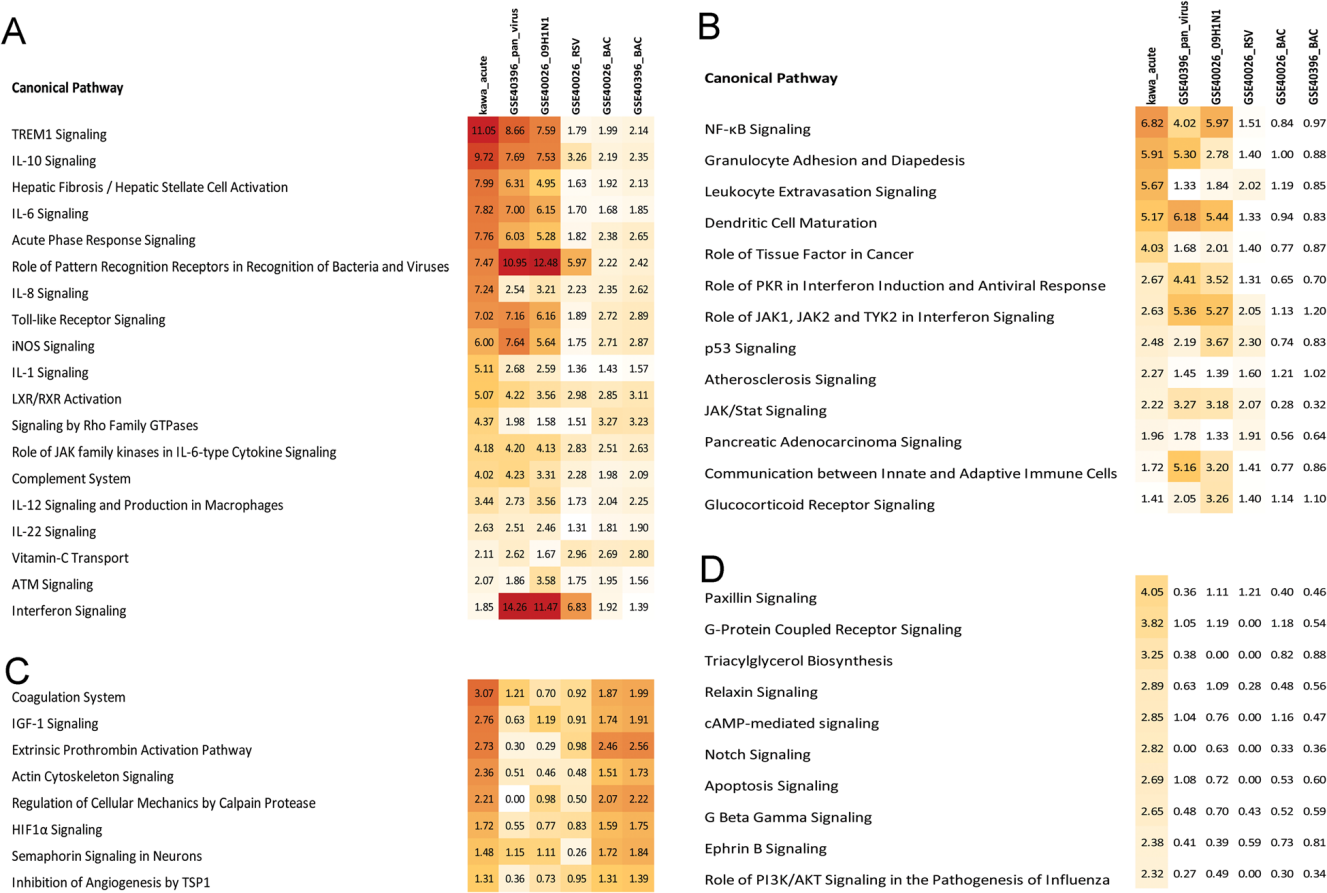
**Comparison of the gene pathway analysis of upregulated pathways in each group of patients determined by the Z test. (A)** Pathways that were upregulated (KD acute vs. conv., other groups vs. healthy controls) and shared among KD, viral, and bacterial infection groups (see Additional file 2: Table S5). **(B)** Pathways that were shared between KD and viral infections. **(C)** Pathways shared by KD and bacterial infections. **(D)** Pathways that were specifically upregulated in KD patients and. These over-represented pathways were identified using IPA database. The numbers in each box represents the -log10 *P* value (BH corrected) identified by Fisher’s exact test. The colors represent the strength of association with pathways with dark red designating the highest and white the lowest level of association.

More and less abundant transcripts in each condition were analyzed using IPA. From the pathway analysis, canonical pathways that were common for all conditions or specific for KD were identified using pathway comparison analysis. From the upregulated transcripts, there were 19 pathways that were over-represented in all disease conditions (Figure 4A). The majority of them were involved in host immune response (Interferon Signaling, TREM1 signaling, Toll-like receptor (TLR) signaling, acute phase response signaling, complement system) and cytokine responses (IL1, IL6, IL8, IL10, IL12, and IL22). Interferon signaling was strongly over-represented in patients infected with viral pathogens, but notably low for the KD subjects (Figure 4A). There were 13 pathways that were common to KD and viral infections including NF-kB signaling, granulocyte adhesion and diapedesis, and dendritic cell maturation (Figure 4B). There were only eight pathways that were shared between KD and bacterial infection patients (Figure 4C), with the most significant being coagulation system, IFG-1 signaling, and extrinsic prothrombin activation pathways. There were 47 pathways that were only over-represented in KD subjects (Figure 4D). Genes related to cell migration and trafficking (paxillin, relaxin, actin, integrins, MMPs) and signal transduction (MAP kinases and phosphatases) were the leading DATs.

**Figure 5:**
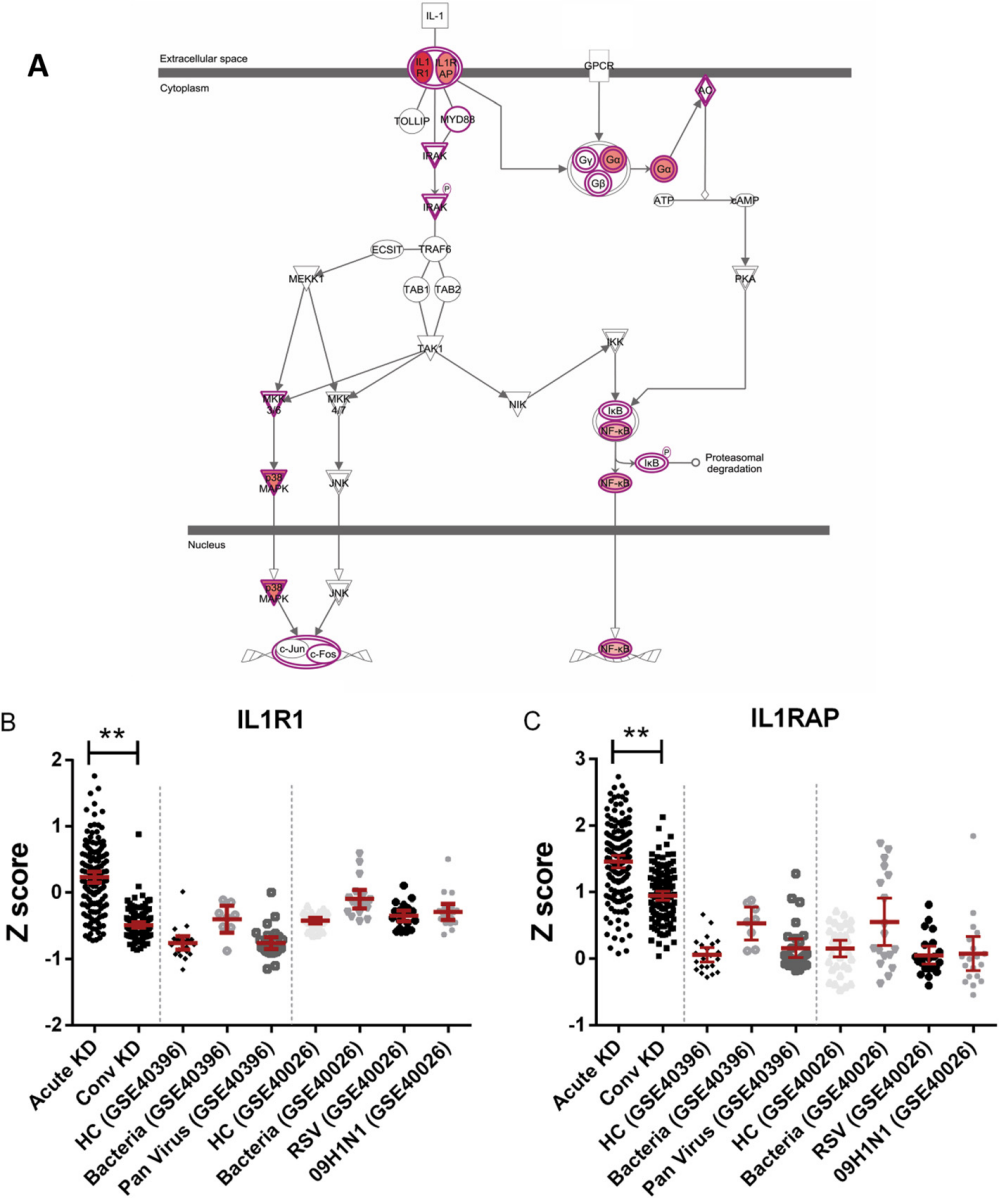
**IL1signaling pathway was the key upregulated pathway in acute KD. (A)** Transcripts involved in IL1 signaling pathways were more abundant in acute KD; DATs between acute and convalescent KD samples are highlighted in red. **(B, C)** IL1R1 and IL1RAP were differentially expressed only in acute vs. convalescent KD but not in other diseases. **P value <0.01.

**Figure 6:**
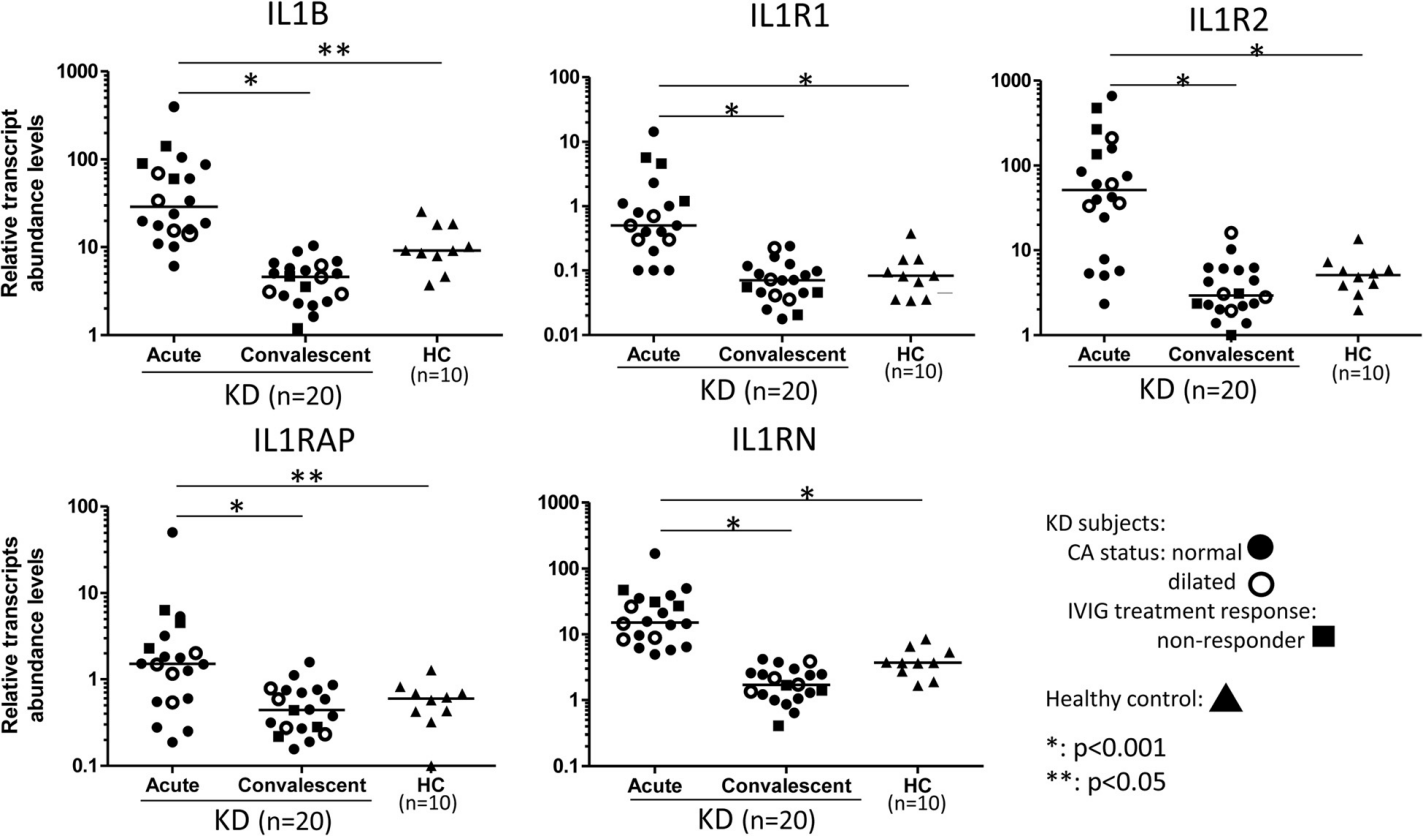
**Transcript abundance levels of IL1B, IL1R1, IL1R2, IL1RAP, and IL1RN were measured in a new cohort of KD subjects (n = 20) and controls (n = 10) by reverse transcriptase-polymerase chain reaction (RTPCR).** The relative abundance of these genes were normalized against the expression level of the house keeping gene (TAF1B). qPCR results showed that these key IL1 genes were highly abundant in acute phase of KD patients in comparison with the convalescent phase and with the controls.

**Figure 7:**
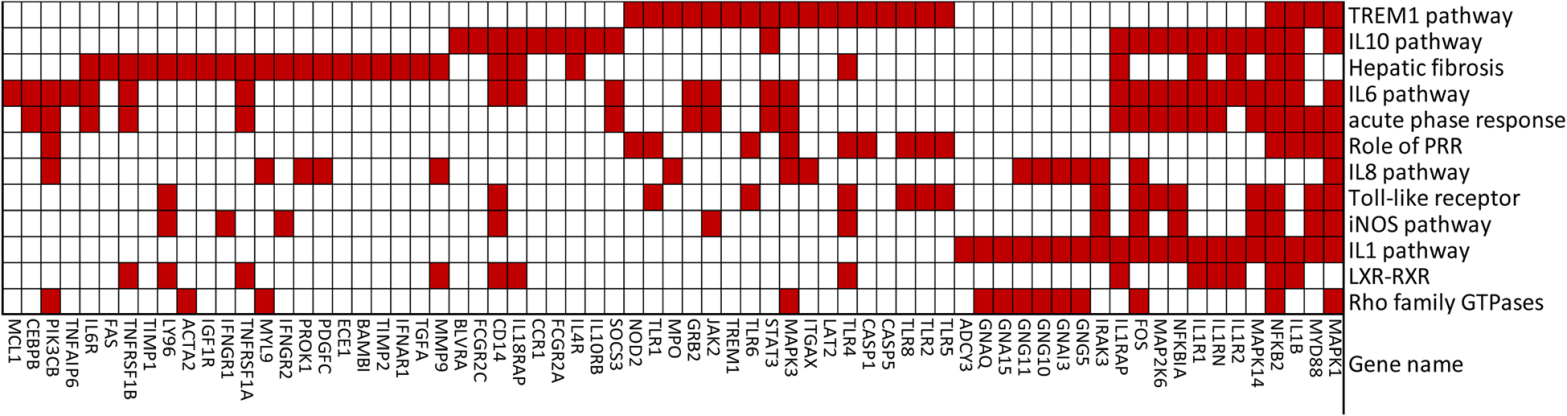
**Significantly upregulated genes in the top 12 pathways comparing acute vs. convalescent Kawasaki disease.** DATs in each pathway were highlighted in red boxes.

Common features of the top three pathways for KD (Trem1 signaling, Hepatic fibrosis, and IL-10 signaling) and the other groups were the abundance of transcripts related to the activation of the Nlrp3 inflammasome, including Il-1 and caspase-1 related transcripts (Figure 5). The key genes in IL1 pathway IL1B, IL1R1, IL1R2, IL1RAP, and IL1RN were validated using qPCR in KD patients (Figure 6). Other key transcripts in these pathways included TLRs, matrix metalloproteinases, NFkB signaling molecules, and IL-10 (Figure 7, Additional file 2: Table S6).

**Figure 8:**
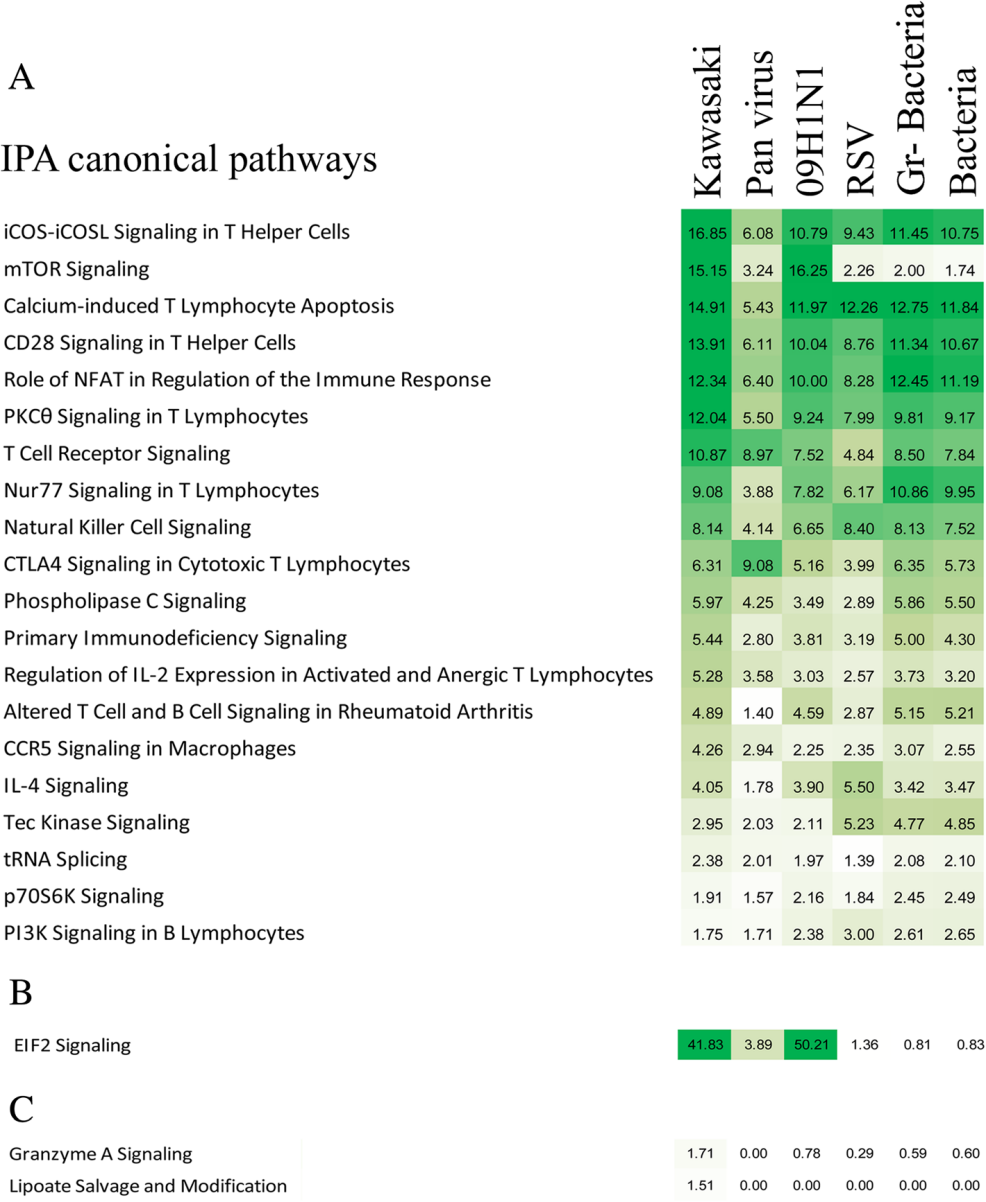
**Comparison of the downregulated pathways.** Similar to the upregulated pathway analysis, these pathways were identified by comparing the gene lists with the IPA database. **(A)** Common pathways those were downregulated in all groups of patients (see Additional file 2: Table S6). **(B)** Downregulated pathways shared by KD and viral infections and **(C)** downregulated KD-specific pathways. The numbers in each box represent the -log10 P value (BH corrected) identified by Fisher’s exact test. The colors represents the strength of association with pathways with dark green designating the highest and white is the lowest level of association.

**Figure 9:**
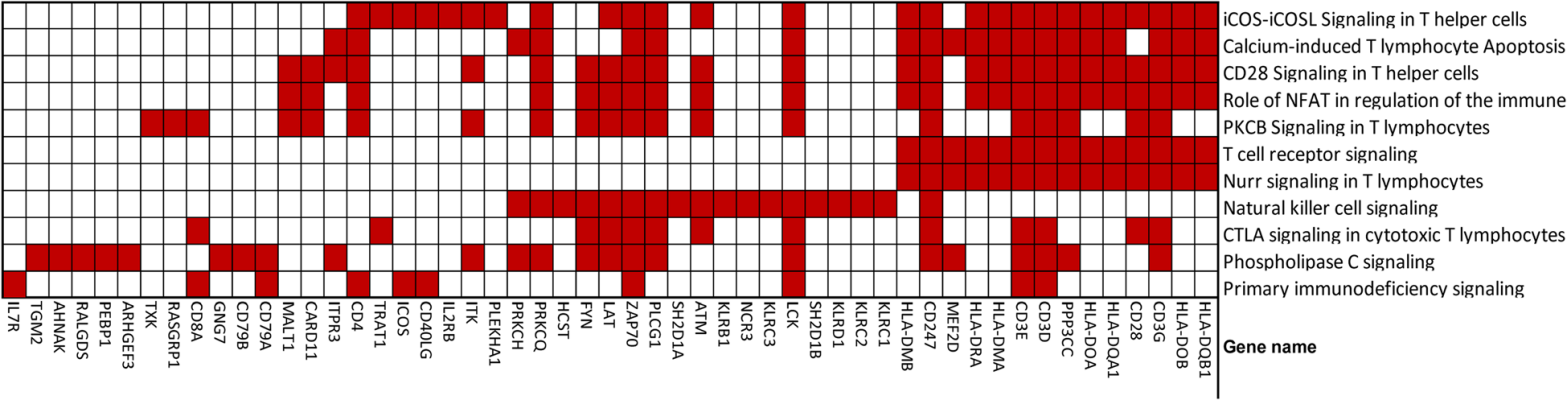
**Significantly downregulated genes in the top 11 pathways comparing acute vs. convalescent Kawasaki disease.** DATs in each pathway were highlighted in red boxes.

We identified 20 pathways that were over-represented in the less abundant transcript group and were shared by all disease conditions (KD acute vs. conv., other groups vs. healthy controls). T cell-related response pathways (iCOS-iCOSL Signaling in T Helper Cells, Calcium-induced T Lymphocyte Apoptosis, CD28 Signaling in T Helper Cells, PKCθ Signaling in T Lymphocytes, and T Cell Receptor Signaling) and NK cell signaling were predominant among these 20 pathways. In addition, IL4, IL2, and C chemokine receptor (CCR) signaling pathways were also prominent (Figure 8A). EIF2 signaling was the only pathway that was downregulated in both KD and virus-infected subjects, with the highest levels of suppression seen for KD and influenza H1N1-infected subjects (Figure 8B). There were no pathways that were uniquely shared between KD and bacteria-infected subjects. Granzyme A signaling and lipoate salvage and modification were the pathways that were specifically downregulated in KD subjects only (Figure 8C). These pathways contain genes related to cytotoxic T cell signaling and apoptosis (Figure 9, Additional file 2: Table S7).

## Discussion

We describe here the similarities and differences between the host transcriptional profiles of acute and convalescent KD subjects and profiles of children with acute bacterial and viral infections. There were several upregulated pathways uniquely over-represented in KD subjects including paxillin signaling, G-protein coupled receptor signaling, triacylglycerol and relaxin signaling. The paxillin signaling pathway, which includes α-smooth muscle actin (ACTA2), actinin, paxillin, talin, and integrins, is important for creating cell to extracellular matrix focal adhesions during myofibroblast transformation and cell migration [18,19]. We also found increased transcript abundance for pathways that oppose myofibroblast formation, including the relaxin signaling pathway, which was uniquely increased in acute vs. convalescent KD. Relaxin prevents cardiac fibroblast-myofibroblast transition via Notch-1-mediated inhibition of TGF-β/Smad3 signaling leading to reduction of α-SMA synthesis and inhibition of extracellular focal adhesion formation [11]. This is of interest because of the proposed role in KD of endothelial/epithelial-to-mesenchymal transition leading to myofibroblast formation. These cells are thought to migrate into the arterial wall and myocardium where they recruit pro-inflammatory cells that contribute to tissue damage [20,21]. Increased expression of genes that block myofibroblast migration may represent an adaptive host response to limit the pathogenic role of these cells.

The most dramatic finding was the marked upregulation in acute KD subjects of pathways involved with the innate immune response and cell migration as compared to subjects in the convalescent phase of KD. Although many of the same pathways were upregulated across all disease groups, the magnitude varied with the KD subjects having greater elevation of selected transcripts in the IL1, IL10, and NF-kB signaling pathways. Pathways with genes involved in IL1 signaling were more abundant in all conditions; however, the key receptors in the pathway were only upregulated in KD patients *(IL1R, IL1RAP)*. IL1R and IL1RAP (IL-1 receptor accessory protein) are expressed on the cell surface where they directly bind IL1, which in turn activates the signaling cascade. All of the top five pathways for KD subjects prominently featured IL-1 signaling molecules including IL-1R, IL-1RAP, IL-1R2 (IL-1 decoy receptor), and IL1RN (IL1 receptor antagonist), thus suggesting that IL-1 signaling plays a critical role in KD pathogenesis. We also observed that while the pro-inflammatory IL1 pathway is activated, the anti-inflammatory IL10 pathway is also activated (STAT3, SOCS3) as a negative regulator of IL-1β synthesis, signaling, and bioavailability [22]. The IL1 receptor antagonist anakinra has been used to treat a severe KD patient who was unresponsive to IVIG [23]. Our observations suggest that the IL-1 signaling pathway is a key mediator in the pathogenesis of KD and may represent an important therapeutic target.

T cell and NK cell-related signatures in the peripheral blood were attenuated across all disease groups with reduced expression of genes involved in antigen presentation through MHC Class II, T cell signaling, and protein synthesis. In contrast to the signature noted for H1N1-infected children in the dataset used in the present study, the signature of decreased protein synthesis for KD subjects was driven by reduced expression of ribosomal proteins without increased expression of genes known to inhibit protein synthesis (for example, EIFAK2) [13]. Both the KD and the H1N1-infected subjects had marked suppression of the EIF2 pathway contributing to the suppression of protein synthesis. Whether the suppression of host protein synthesis in KD subjects is an adaptive response to reduce pathogen-directed protein synthesis or whether this represents a pathogenic suppression of host protein synthesis by the ‘KD agent’ is unknown. What is clear is that transcript abundance of genes critical for protein synthesis is markedly reduced in acute KD subjects.

Variation in global gene expression patterns related to IVIG response has been reported by several groups [9,24–27]. In our study of the largest sample size ever reported, we showed that patients who responded to IVIG treatment had more abundant transcripts in pathways involved in T and NK cell responses. Transcripts that were involved in the majority of these pathways were *CD3E, CD4, TNFRSF3B, SERPINA1, MME,* and *IRF4*. Among the top DATs when stratified by the magnitude of the fold-difference between acute blood samples from IVIG responsive and resistant subjects were MMP-8, CEACAM1, and PFKB2. Transcripts for both MMP-8 and CEACAM1 were elevated in IVIG-resistant KD subjects in three previous studies [10,24,26]. MMP8 or neutrophil collagenase may be secreted by neutrophils infiltrating into the arterial wall that contribute to the persistence of inflammation in IVIG-resistant KD patients [28,29]. CEACAM1 is expressed on the surface of endothelial cells, lymphocytes, and myeloid cells, and acts as an activation-induced co-inhibitory receptor on T cells [30]. Expression of CEACAM1 on activated T cells in KD patients may represent a compensatory mechanism to decrease the pro-inflammatory response. Alternatively, increased expression of CEACAM1 on neutrophils is associated with delayed apoptosis, which may be related to the observed persistence of inflammation in KD patients failing to respond to IVIG [8,13]. PFKB2 has not been previously reported in association with KD, but its role as an intracellular regulator of glycolysis in cardiomyocytes and its increased expression in the right ventricle of children with Tetralogy of Fallot and right ventricular pressure overload is intriguing [31]. Myocarditis is a universal feature of acute KD and one could speculate that patients with IVIG-resistance who have a global increase in inflammation may also have more intense myocardial inflammation leading to altered glycolysis in cardiomyocytes [32].

Of the genes differentially expressed between the CAA and normal CA groups, CYP26B1 had a biologically plausible link with the inflammatory process of KD. Cyp26b1 is a member of the cytochrome P450 system that is expressed in many cell types including vascular smooth muscle cells and T cells in which it acts as a negative regulator of retinoic acid signaling. Retinoic acid has widespread vascular effects including inhibition of intimal proliferation and effects on differentiation of naïve T cells. Depending on the cytokine milieu, retinoic acid can stimulate the differentiation of naïve T cells toward either a regulatory (Treg) or inflammatory (Th17) phenotype [33]. KD subjects with CAA had decreased expression of Cyp26b, which would result in increased retinoic acid signaling and potentially increases in pro-inflammatory CD4+ Th17 cells. Functional polymorphisms in Cyp26b1 have been described that modulate gene expression and if the association of reduced Cyp26b1 gene expression is validated in independent cohorts of CAA vs. normal CA cohorts, then genotyping for these variants may contribute to our understanding of susceptibility to CAA. Gene expression for the skin aspartic acid protease (SASPase) gene was also suppressed in KD subjects with CAA. This protease plays a key role in profilaggrin-filaggrin processing and reduced expression in mice leads to an eczema phenotype [34]. It is of interest that eczema is over-represented among patients with KD and perhaps genetic variants in this gene may contribute to this phenotype [35,36]. However, the association with CAA is unclear as expression is claimed to be limited to the skin [37]. Suppression of NKX3-1, a homeobox-containing transcription factor that is regulated by TNFα and IL1β, was also observed in CAA KD subjects. One target of NKX3-1 is vascular endothelial growth factor (VEGF), a protein that stimulates lymph node lymphangiogenesis [38]. Hyperplasia of lymph nodes that drain the posterior pharynx is a prominent feature of acute KD. Reduced levels of NKX3-1 would be expected to increase VEGFC signaling and lead to lymph node enlargement, although the relationship with CAA is unclear. TRANK1 expression was also suppressed in CAA KD although little is known about the function of this gene.

Several previous observations regarding KD gene expression are supported by the data in the present study. The muted signature for interferon signaling was in sharp contrast to the expression profiles for viral-infected subjects, especially those infected with H1N1 [39,40]. The low abundance of interferon and interferon-induced transcripts was also observed in a study of gene expression profiles in acute KD subjects compared to subjects with adenovirus infection [16]. The observation was confirmed by RT-PCR of the interferon-inducible genes MX1, ISG15, and LY6E in an independent cohort of KD subjects [10]. Notable differences in the DAT patterns in KD observed in the present study compared to previous reports may be due to both the larger samples size as well as the correction for cell numbers. Polycythemia vera 1 (CD177), which is expressed by activated neutrophils and has been previously reported as a leading DAT in KD, was not among the top DATs in our study [27]. This is likely due to the large difference in absolute neutrophil count between acute and convalescent blood samples from KD subjects, which was accounted for in our analysis with the GLM method. Previous studies did not correct for absolute neutrophil count.

We recognize several strengths and weaknesses to the present study. We have created the largest database of DATs in KD ever assembled and this valuable resource is now available to other investigators to mine for data to address other questions related to KD. It was beyond the scope of the present study to perform RT-PCR assays for DAT validation for all the potential targets or to measure serum levels of specific proteins. The results presented here must therefore be viewed in the spirit of generating hypotheses about novel pathways and proteins that must be validated in independent cohorts. However, the large sample size gives good statistical confidence in the results obtained.

## Conclusion

In conclusion, our study of DATs in acute and convalescent KD whole blood samples revealed the importance of the IL-1 signaling pathway and a prominent signature of innate immunity and cell migration in the acute phase of the illness. Pathways predicted to both increase myofibroblast transformation and migration and to oppose myofibroblast formation were also upregulated in the acute disease. Signatures for protein synthesis and T and NK cells were markedly depressed in acute KD. Important differences from other infectious diseases were the attenuation of an interferon signature in KD vs. viral infections. IVIG responders had increased transcript abundance for genes associated with T and NK cells, while IVIG-resistant subjects had increased transcripts for genes association with neutrophil infiltration and apoptosis. A novel finding related to the host response to IVIG was the association of PFKB2, a master regulator of cardiomocyte glycolysis, with IVIG resistance. CAA was associated with transcripts related to eczema and lymph node hyperplasia. Most importantly, CAA was associated with decreased transcript abundance of CYP26b1, a negative regulator of retinoic acid signaling, Opportunities for translation of these observations include the use of agents that block the IL-1 signaling pathway and agents that stimulate the retinoic acid signaling pathway toward T cell regulation.

## Additional files

Additional file 1: Figure S1. Diagram shows the number of subjects used for the study. There were 171 subjects with KD were recruited to the study **(A)**. Expression data from children with the same age group who were acutely infected with either viral or bacterial pathogens were retrieved from two published studies. Data from 57 subjects were downloaded from GSE40396 study **(B)** and data from 82 subjects were downloaded from study GSE42026. Additional file 2: Table S1. Clinical characteristics and laboratory values at acute and convalescent time points for KD subjects. **Table S2.** Clinical characteristics and laboratory values for IVIG-responder and IVIG-resistant subjects. **Table S3.** Top five DATs in acute blood samples from IVIG-responsive vs. IVIG-resistant KD subjects. Genes are ordered based on Z score difference with negative scores indicating genes with reduced expression in IVIG-responsive subjects. **Table S4.** Clinical characteristics and laboratory values for KD subjects with normal and aneurysmal coronary arteries. **Table S5.** Clinical characteristics and laboratory values of the new KD patients and controls used for qPCR assay. **Table S6.** Significantly upregulated genes in each pathway comparing acute vs. convalescent Kawasaki disease blood samples listed in order of descending *P* values. Genes highlighted in yellow were significant in at least two separate pathways. **Table S7.** Significantly downregulated genes in each pathway comparing acute vs. convalescent Kawasaki disease blood samples listed in order of descending *P* values. Genes highlighted in yellow were significant in at least two separate pathways.

## Abbreviations

CAA: Coronary artery aneurysm
DAT: Differentially expressed transcripts
GLM: Generalized linear model
IPA: Ingenuity pathway analysis
IVIG: Intravenous immunoglobulin
KD: Kawasaki disease
