# Supplementary figures and images for: Global gene expression profiling identifies new therapeutic targets in acute Kawasaki disease
Source: Genome Med. 2014 Nov 20;6(11):541. doi: 10.1186/s13073-014-0102-6 (PMC4279699; doi:10.1186/s13073-014-0102-6)

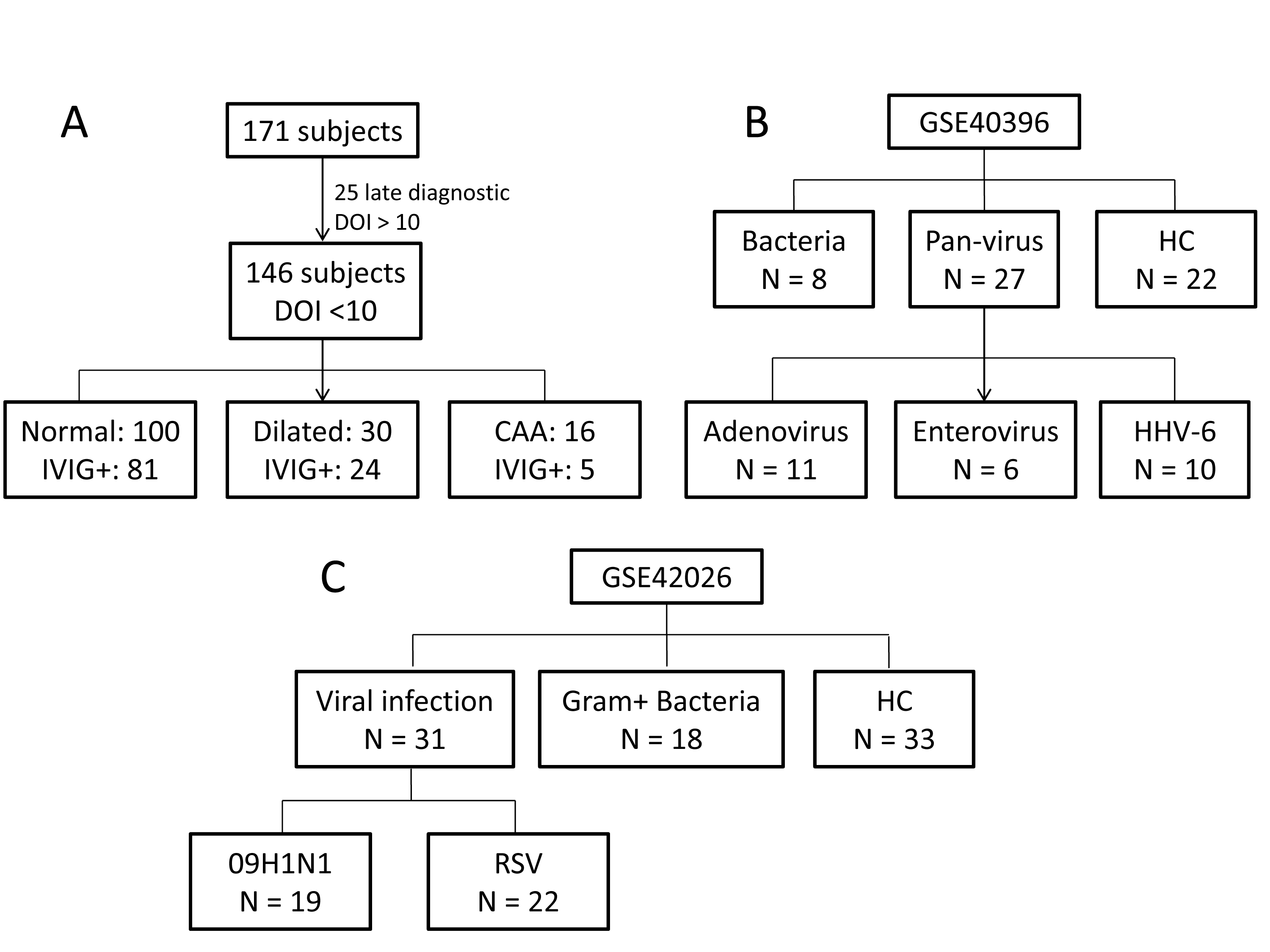

Supplement: Additional file 1: Figure S1. — Diagram shows the number of subjects used for the study. There were 171 subjects with KD were recruited to the study (A). Expression data from children with the same age group who were acutely infected with either viral or bacterial pathogens were retrieved from two published studies. Data from 57 subjects were downloaded from GSE40396 study (B) and data from 82 subjects were downloaded from study GSE42026. [file 13073_2014_102_MOESM1_ESM.tiff]
