# Supplementary material for: Global gene expression profiling identifies new therapeutic targets in acute Kawasaki disease
Source: Genome Med. 2014 Nov 20;6(11):541. doi: 10.1186/s13073-014-0102-6 (PMC4279699; doi:10.1186/s13073-014-0102-6)
Supplement: Additional file 2: Table S1. — Clinical characteristics and laboratory values at acute and convalescent time points for KD subjects. Table S2. Clinical characteristics and laboratory values for IVIG-responder and IVIG-resistant subjects. Table S3. Top five DATs in acute blood samples from IVIG-responsive vs. IVIG-resistant KD subjects. Genes are ordered based on Z score difference with negative scores indicating genes with reduced expression in IVIG-responsive subjects. Table S4. Clinical characteristics and laboratory values for KD subjects with normal and aneurysmal coronary arteries. Table S5. Clinical characteristics and laboratory values of the new KD patients and controls used for qPCR assay. Table S6. Significantly upregulated genes in each pathway comparing acute vs. convalescent Kawasaki disease blood samples listed in order of descending P values. Genes highlighted in yellow were significant in at least two separate pathways. Table S7. Significantly downregulated genes in each pathway comparing acute vs. convalescent Kawasaki disease blood samples listed in order of descending P values. Genes highlighted in yellow were significant in at least two separate pathways. [file 13073_2014_102_MOESM2_ESM.docx]

Additional file 2

TableS1. Clinical characteristics and laboratory values at acute and convalescent time points for KD subjects.

|  | **Acute**  **(n = 146)** | **Convalescent**  **(n = 131)** | ***P* value^a^** |
| --- | --- | --- | --- |
| Male, n (%) | 86 (59) | 79 (60) | -- |
| Age, months | 33 (4 - 182)^b^ | - | -- |
| Illness Day, days^c^ | 6 (2 - 10) | 51 (19 – 2,230) | -- |
| Zworst, SD units^d^ | 1.7 (-0.6 - 18.3) | | -- |
| WBC, x 10^3^/mm^3^ | 14 (3.2 - 33.9) | 8 (3.7 - 17.2) | <0.001 |
| % Neutrophils | 54 (2 - 82) | 37 (7 - 69) | <0.001 |
| % Bands | 14 (0 - 75) | 1 (0 - 8) | <0.001 |
| % Lymphocytes | 20 (2 - 82) | 51 (19 - 82) | <0.001 |
| % Monocytes | 6 (0 - 17) | 6 (1 - 18) | -- |
| % Eosinophils | 2 (0 - 35) | 3 (0 - 32) | <0.05 |
| Absolute neutrophil count, x 10^3^/mm^3^ | 9.4 (1 - 27.5) | 2.9 (0.8 – 7.1) | <0.001 |
| Hemoglobin z-score, SD units | -1.2 (-7.0 - 2.4) | -0.2 (-3.1 - 3.0) | <0.001 |
| Platelet count, x 10^3^/mm^3^ | 404 (89 - 918) | 393 (238 - 690) | -- |
| ESR, mm/h | 61 (5 - 140) | 11 (1 - 36) | <0.001 |
| CRP, mg/dL | 8.1 (0.3 - 45.2) | 0.3 (0 - 0.9) | <0.001 |
| ALT, IU/L | 44 (3 - 725) | 53 (49 - 88) | -- |
| GGT, IU/L | 38 (7 - 432) | N/A | N/A |

^a^*P* values calculated by Mann-Whitney U test.

^b^Values are presented as median (range).

^d^Illness day 1= first calendar day of fever.

^d^Zworst= highest value of RCA and LAD coronary artery Z score at any time point normalized for body surface area.

ALT: alanine aminotransferase (normal range: 10-25 IU/L), CRP: C-reactive protein (normal range: <0.5 mg/dl), ESR: erythrocyte sedimentation rate (normal range: 0-15 mm/h), GGT: gamma-glutamyltransferase (normal range: 10-22 IU/L), Hemoglobin Z score: hemoglobin concentration normalized for age, N/A: not available, WBC: white blood count (normal range: 4-12 x 10^3^/mm^3^)

Table S2. Clinical characteristics and laboratory values for IVIG-responder and IVIG-resistant subjects.

|  | **IVIG responder**  **(n = 110)** | **IVIG resistant**  **(n = 30)** | ***P* value^a^** |
| --- | --- | --- | --- |
| Male, n (%) | 65 (59) | 17 (57) |  |
| Median age (range), months | 34.5 (4 - 182) | 28.5 (4 - 179) |  |
| Median illness day (range), days^b^ | 6 (2 - 10) | 5 (2 - 10) |  |
| Ethnicity, n (%) |  |  |  |
| Asian | 20 (18) | 5 (17) |  |
| Caucasian | 28 (25) | 7 (23) |  |
| African American | 4 (4) | 1 (3) |  |
| Hispanic | 25 (23) | 9 (30) |  |
| Mixed | 33 (30) | 8 (27) |  |
| CA status, n (%) |  |  |  |
| Normal | 81 (74) | 18 (60) |  |
| Dilated | 24 (22) | 5 (17) |  |
| Aneurysm | 5 ( 4) | 7 (23) |  |
| Median Zworst (range), SD units | 1.7 (-0.6 - 18.3) | 1.6 (0.2 - 15.1) |  |
| Lab data, median (range) |  |  |  |
| WBC, x 10^3^/mm^3^ | 14.5 (3.2 - 33.9) | 13.6 (6.9 - 30.5) |  |
| % Neutrophil | 56 (7 - 82) | 50 (2 - 73) | <0.05 |
| % Bands | 14 (0 - 55) | 18 (0 - 75) | <0.05 |
| % Lymphocyte | 20 (2 - 82) | 18.5 (2 - 46) |  |
| % Monocyte | 6 (0 - 17) | 5.5 (1 - 15) |  |
| % Eosinophil | 2 (0 - 35) | 1 (0 - 19) |  |
| Absolute neutrophil count, x 10^3^/mm^3^ | 9.4 (1.0 - 27.5) | 9.8 (3.2 - 25.9) |  |
| Hemoglobin z-score, SD units | -1.2 (-7.0 - 2.3) | -1.3 (-4.2 - 2.4) |  |
| Platelet count, 10^3^/mm^3^ | 410 (89 - 918) | 378.5 (114 - 706) |  |
| ESR, mm/h | 64 (16 - 140) | 58 (12 - 140) |  |
| CRP, mg/dL | 8 (0.3 - 39.3) | 12.0 (1.2 - 45.2) |  |
| ALT, IU/L | 32 (3 - 725) | 70 (12 - 338) |  |
| GGT, IU/L | 32 (7 - 432) | 89 (10 - 389) |  |

^a^*P* values were calculated by Mann-Whitney U test.

^b^Illness day 1: first calendar day of fever.

ALT: alanine aminotransferase (normal range: 10-25 IU/L), CRP: C-reactive protein (normal range: <0.5 mg/dl), ESR: erythrocyte sedimentation rate (normal range: 0-15 mm/h), GGT: gamma-glutamyltransferase (normal range: 10-22 IU/L), Hemoglobin Z score: hemoglobin concentration normalized for age, WBC: white blood count (normal range: 4-12 x 10^3^/mm^3^)

Table S3. Top five DATs in acute blood samples from IVIG-responsive vs. IVIG-resistant KD subjects. Genes are ordered based on Z score difference with negative scores indicating genes with reduced expression in IVIG-responsive subjects.

Table S4. Clinical characteristics and laboratory values for KD subjects with normal and aneurysmal coronary arteries.

|  | Normal CA  (n = 100) | Aneurysm  (n = 16) | *P* value^a^ |
| --- | --- | --- | --- |
| Male, n (%) | 55 (55) | 11 (69) |  |
| Median age (range), months | 36.5 (4 - 182) | 16.0 (4-129) |  |
| Median illness day (range), days^b^ | 6 (2 - 10) | 6 (3-10) |  |
| Ethnicity, n (%) |  |  |  |
| Asian | 16 (16) | 5 (31.5) |  |
| Caucasian | 27 (27) | 2 (12.3) |  |
| African American | 4 (4) | 2(12.3) |  |
| Hispanic | 20 (20) | 5 (31.5) |  |
| Mixed | 33 (33) | 2 (12.3) |  |
| Median Zworst (range), SD units | 1.3 (-0.6 - 2.4) | 6.9 (3.4 - 18.3) | <0.001 |
| Lab data, median (range) |  |  |  |
| WBC, x 10^3^/mm^3^ | 14.4 (3.2 - 33.9) | 13.9(7.6 - 26.4) |  |
| % Neutrophil | 56 (7 - 82) | 44 (2 - 74) | <0.05 |
| % Bands | 14 (0 - 64) | 22 (0 - 75) |  |
| % Lymphocyte | 19 (2 - 82) | 25 (4 - 46) |  |
| % Monocyte | 6 (0 - 17) | 5 (2 - 13) |  |
| % Eosinophil | 2 (0 - 35) | 1 (0 - 11) |  |
| Absolute neutrophil count, x 10^3^/mm^3^ | 9.7 (1.0 - 26.2) | 7.8 (4.3-18.9) |  |
| Hemoglobin z-score, SD units | -1.2 (-7.0 – 2.0) | -1.2 (-4.1 - 2.4) |  |
| Platelet count, 10^3^/mm^3^ | 398 (104 - 918) | 401 (114 - 651) |  |
| ESR, mm/h | 63 (5 - 140) | 57 (12 - 140) |  |
| CRP, mg/dL | 6.8 (0.3 - 45.2) | 10.4 (0.3 - 38.1) |  |
| ALT, IU/L | 41 (3 - 725) | 74 (14 - 338) |  |
| GGT, IU/L | 30 (7 - 432) | 100 (15 - 251) | <0.05 |

^a^*P* values were calculated by Mann-Whitney U test for continuous.

^b^Illness day 1: first calendar day of fever.

ALT: alanine aminotransferase (normal range: 10-25 IU/L), CRP: C-reactive protein (normal range: <0.5 mg/dl), ESR: erythrocyte sedimentation rate (normal range: 0-15 mm/h), GGT: gamma-glutamyltransferase (normal range: 10-22 IU/L), Hemoglobin Z score: hemoglobin concentration normalized for age, WBC: white blood count (normal range: 4-12 x 10^3^/mm^3^)

Table S5. Clinical characteristics and laboratory values of the new KD patients and controls used for qPCR assay.

| **Variable** | **KD patients (n = 20)** | **Healthy controls (n = 10)** |
| --- | --- | --- |
| Age, months | 36.9 (5.8 - 136.8)^a^ | 22.8 (4.6 - 68.5) |
| Male, n (%) | 8 (40) | 4 (40) |
| Illness day at sample collection | 5 (3 - 9) | NA |
| **CA status:** | 16 (80) | NA |
| Normal, n (%) |  |  |
| Dilated, n (%) | 4 (20) | NA |
| Zworst | 1.55 (0.6 -4.1) | NA |
| IVIG-resistant, n (%) | 3 (15) | NA |
| White blood count (x 10^3^/mm^3^) | 12.9 (4.4 - 35) | NA |
| % Neutrophils | 49.5 (20 - 79) | NA |
| Absolute neutrophil count (x 10^3^/mm^3^) | 8.7 (2.2 - 33.6) | NA |
| Absolute band count | 0.9 (0 - 8.7) | NA |
| % Lymphocytes | 23 (2 - 43) | NA |
| Acute Hemoglobin z-score | -1.2 (-3.3 - 0.4) | NA |
| Platelet count (x 10^3^/mm^3^) | 320 (133 - 611) | NA |
| Acute ESR (mm/h) | 61 (23 - 104) | NA |
| Acute C-reactive protein (mg/dL) | 7.2 (3.2 - 31.1) | NA |

^a^All the values were presented in median (range).

**Table S6. Significantly upregulated genes in each pathway comparing acute vs. convalescent Kawasaki disease blood samples listed in order of descending *P* values. Genes highlighted in yellow were significant in at least two separate pathways.**

Table S7. Significantly downregulated genes in each pathway comparing acute vs. convalescent Kawasaki disease blood samples listed in order of descending *P* values. Genes highlighted in yellow were significant in at least two separate pathways.
